# The efficacy of a home-use metabolic device (Lumen) in response to a short-term low and high carbohydrate diet in healthy volunteers

**DOI:** 10.1080/15502783.2023.2185537

**Published:** 2023-03-02

**Authors:** Justin Roberts, Dirk Dugdale-Duwell, Joseph Lillis, Jorge Marques Pinto, Ash Willmott, Shlomo Yeshurun, Merav Mor, Tjeu Souren

**Affiliations:** a Cambridge Centre for Sport and Exercise Sciences (CCSES), School of Psychology and Sport Science, Anglia Ruskin University, Cambridge, UK; b Occupational and Environmental Physiology Group, Centre for Sport Exercise and Life Sciences, Coventry University, Coventry, UK; c MetaFlow Ltd, Tel Aviv, Israel; d Utrecht University Medical Center, Utrecht, The Netherlands; e School of Human Biology, Maastricht University, Maastricht, The Netherlands

**Keywords:** Lumen, respiratory, dietary, nutrition, metabolism, carbohydrate

## Abstract

**Background:**

Based on stoichiometric assumptions, and real-time assessment of expired carbon dioxide (%CO_2_) and flow rate, the Lumen device provides potential for consumers/athletes to monitor metabolic responses to dietary programs outside of laboratory conditions. However, there is a paucity of research exploring device efficacy. This study aimed to evaluate Lumen device response to: i) a high-carbohydrate meal under laboratory conditions, and ii) a short-term low- or high-carbohydrate diet in healthy volunteers.

**Methods:**

Following institutional ethical approval, 12 healthy volunteers (age: 36 ± 4 yrs; body mass: 72.1 ± 3.6 kg; height: 1.71 ± 0.02 m) performed Lumen breath and Douglas bag expired air measures under fasted laboratory conditions and at 30 and 60 min after a high-carbohydrate (2 g·kg^−1^) meal, along with capilliarized blood glucose assessment. Data were analyzed using a one-way ANOVA, with ordinary least squares regression used to assess the model between Lumen expired carbon dioxide percentage (L%CO_2_) and respiratory exchange ratio (RER). In a separate phase, 27 recreationally active adults (age: 42 ± 2 yrs; body mass: 71.9 ± 1.9 kg; height: 1.72 ± 0.02 m) completed a 7-day low- (~20% of energy intake [EI]; LOW) or high-carbohydrate diet (~60% of EI; HIGH) in a randomized, cross-over design under free-living conditions. L%CO_2_ and derived Lumen Index (L_I_) were recorded daily across morning (fasted and post-breakfast) and evening (pre/post meal, pre-bed) periods. Repeated measures ANOVA were employed for main analyses, with Bonferroni post-hoc assessment applied (*P* ≤ 0.05).

**Results:**

Following the carbohydrate test-meal, L%CO_2_ increased from 4.49 ± 0.05% to 4.80 ± 0.06% by 30 min, remaining elevated at 4.76 ± 0.06% by 60 min post-feeding (*P* < 0.001, η_p_^2^ = 0.74). Similarly, RER increased by 18.1% from 0.77 ± 0.03 to 0.91 ± 0.02 by 30 min post-meal (*P* = 0.002). When considering peak data, regression analysis demonstrated a significant model effect between RER and L%CO_2_ (F = 5.62, *P* = 0.03, R^2^ = 0.20). Following main dietary interventions, no significant interactions (diet × day) were found. However, main diet effects were evident across all time-points assessed, highlighting significant differences for both L%CO_2_ and L_I_ between LOW and HIGH conditions (*P* < 0.003). For L%CO_2_, this was particularly noted under fasted (4.35 ± 0.07 vs. 4.46 ± 0.06%, *P* = 0.001), pre-evening meal (4.35 ± 0.07 vs. 4.50 ± 0.06%, *P* < 0.001), and pre-bed time-points (4.51 ± 0.08 vs. 4.61 ± 0.06%, *P* = 0.005).

**Conclusion:**

Our findings demonstrated that a portable, home-use metabolic device (Lumen) detected significantly increased expired %CO_2_ in response to a high-carbohydrate meal, and may be useful in tracking mean weekly changes to acute dietary carbohydrate modifications. Additional research is warranted to further determine the practical and clinical efficacy of the Lumen device in applied compared to laboratory settings.

## Introduction

Nutritional intake is an essential component influencing exercise adherence and aerobic/anaerobic sports performance [1–7]. As part of this, metabolic responses to dietary intake can provide strategic advantages when planning and undertaking exercise programs [8]. The importance of individualized dietary advice has been increasingly recognized, including day-to-day tailored recommendations before, during, and after training and/or competition [3]. Estimates of metabolic responses under resting (fed/fasted) and exercise conditions are typically measured using indirect calorimetry, notably the use of Douglas bag expired air collection [9,10], through to ventilated hood or breath-to-breath analyzers [10–15]. Through assessment of expired fractional gas measures under controlled laboratory conditions [15], metabolic rate and relative contribution of energy substrates (notably fat and carbohydrate oxidation rates) can be estimated from stoichiometric equations [15–20] or extrapolated from the respiratory quotient (RQ)/respiratory exchange ratio (RER) [21]. Estimation of macronutrient contribution to energy demands is important for evaluating metabolic change and/or metabolic flexibility in relation to dietary or exercise-based interventions [22–25]. However, accurate determination of metabolic patterns typically involves controlled laboratory conditions, as well as expensive analytical equipment [13,26–28].

Moreover, while laboratory-based studies tend to report pre-to-post dietary intervention effects, it is challenging to accurately quantify non-laboratory-based metabolic measures to quantify the metabolic effects of dietary programs. Home-use devices such as glucose monitors and cardiovascular trackers are becoming more available and, with technological advancements, are becoming more cost-effective [29]. However, there are currently limited market resources to quantify expired air analysis outside of laboratory conditions (i.e. at home or other field settings). Therefore, the potential to accurately assess or track substrate utilization away from the laboratory may be beneficial in supporting nutrition or exercise-based research (e.g. participant adherence to dietary interventions) or from a consumer self-monitoring perspective (e.g. application of a personalized nutrition approach, or periodized dietary preparations for an event). One such device (Lumen, MetaFlow Ltd.) currently available to consumers is marketed as the ‘first hand-held, portable device to accurately measure metabolism’ (www.lumen.me). Based on real-time assessment of expired percentage of carbon dioxide (%CO_2_) and flow rate after a breath-hold procedure, the Lumen device is proposed to support monitoring for weight loss programs and longer-term metabolic flexibility, as well as support individual responses to tailored dietary strategies. Based on stoichiometric assumptions, a lower RER (i.e. volume of expired CO_2_ [VCO_2_] per minute divided by the volume of inspired oxygen [VO_2_] per minute) of ~0.7 indicates the prevalence of fat oxidation to total energy contribution. In contrast, a raised RER value approaching ~1.0 indicates greater dominance from total carbohydrate oxidation [30,31].

Whilst the Lumen device only measures %CO_2_, previous validation work has been undertaken in comparison to laboratory-based assessment of RER [32]. Following a 150 g total glucose load, both RER and Lumen %CO_2_ (L%CO_2_) significantly increased, with regression analysis supporting the agreement between an acute 0.09 unit increase in RER corresponding with a 0.28% increase in L%CO_2_ [32]. Therefore, the Lumen device could provide indirect estimates of substrate use [33], and potentially be used to track changes in dietary (i.e. carbohydrate) intake [34]. However, assessment of the Lumen device under free-living conditions is warranted to understand device efficacy, particularly in response to acute dietary changes (e.g. a reduction in total carbohydrate intake or carbohydrate loading). This could facilitate its use in wider research and clinical settings, benefit sport and exercise applications, and support consumer guidance. This project therefore involved two aims: i) to undertake an independent assessment of the Lumen device under controlled laboratory conditions in relation to a standardized test meal; and ii) to investigate the efficacy of the Lumen device in tracking %CO_2_ responses to an acute low- and high-carbohydrate diet in healthy volunteers.

## Materials and methods

### Ethical approval

This project employed a two-phase approach encompassing a laboratory-based device assessment and a separate dietary intervention study. For both phases, institutional ethical approval was obtained from the School of Psychology and Sports Science Research Ethics Panel, Anglia Ruskin University (Ethical approval number: SREP/SES_Staff_19-21) and was conducted in accordance with the Declaration of Helsinki (2013).

### Phase 1: laboratory-based device assessment

#### Study participants and eligibility criteria

Following *a priori* power calculation (G*power3, Dusseldorf, Germany [35]; using α = 0.05; 1 − β = 0.80, based on observed L%CO_2_ responses to a test meal [32]), a minimum sample size of 10 was determined. Participants were required to meet specific eligibility criteria for this phase, including: no known history of metabolic disorders, cardiovascular disease, or hypertension; no recent illnesses or viral infections (including Covid-19); no use of medication or participating in any current diet programs. All participants were considered generally healthy, with a body mass index (BMI) of <29.9 kg·m^2^, and were actively engaged in recreational exercise weekly (~3–5 sessions per week). Importantly, all participants were required to have access to a smartphone (iOS or Android) for the purposes of Lumen mobile application accessibility. Before study inclusion, participants attended a full study briefing and provided written informed consent. From initial recruitment, 12 participants (5 male, 7 female) satisfactorily completed this phase (mean ± standard error [SE]: age: 36 ± 4 yrs; body mass: 72.1 ± 3.6 kg; height: 1.71 ± 0.02 m; BMI: 24.6 ± 1.2 kg·m^2^).

#### Experimental protocol

All participants were initially provided with an individual Lumen device with study-specific login details and guided instructions for use. An initial 7-day period was allocated to allow participant familiarization with the device and the specific breath maneuver, and synchronization of the Lumen software to individual daily breath responses. The device utilizes a single breath measure (after a standardized seated rest period) involving a deep inhalation with a 10 s breath hold followed by a flow-rate controlled exhalation procedure. Based on the assumption that under resting state conditions individual oxygen consumption (O_2_) should remain relatively constant, metabolic fluctuations should therefore reflect CO_2_ changes in expired air. Device reliability was derived from duplicate %CO_2_ measures within <0.2%. If the error margin exceeded 0.2%CO_2_, users were required to rest a further 5 min before repeating the procedure to ensure stable reliability. A Lumen Index (L_I_) from 1 to 5 is derived from the application software based on individual updated L%CO_2_ range (lowest to highest), in alignment with prior validation research [32]. An L_I_ of 1 indicates the lowest assumed RER based on L%CO_2_ regression agreement with RER (i.e. greater relative fat oxidation), and 5 indicates a higher assumed RER (hence greater relative carbohydrate utilization), with remaining values based on quintile distribution of the individual L%CO_2_ range.

Following device habituation, participants attended the Human Physiology Laboratory in a rested state (avoiding exercise in the pre-24 h period) and having completed a standardized overnight fasting period (~10–12 h). Upon arrival, participants were assessed for body mass (Tanita SC-330ST, Amsterdam, The Netherlands) and height (Seca CE123, Hamburg, Germany), before resting in a comfortable supine position for 10 min in a thermoneutral environment (20.6 ± 0.9°C, 61 ± 5% humidity, 758.3 ± 2.8 mmHg). Participants then performed duplicate seated Lumen measures as guided by the mobile application platform. On completion of the Lumen measures, participants remained seated whilst duplicate expired air samples were collected using the Douglas bag method, and analyzed for percentage O_2_ and CO_2_ using a Servomex MINIMP 5200 gas analyzer (Servomex Group Ltd, Crowborough, UK; gas calibration accepted with a <0.2% error margin, and flow rate/volume calibration accepted with a <1.0% error margin). Total Douglas bag volume was measured using a Harvard dry gas meter (Harvard Apparatus, Holliston, USA), with sample temperature recorded during volume measurement. Estimates of VO_2_ and VCO_2_ were corrected against ambient environmental conditions. A 20 μL capillarized blood sample was then collected for analysis of glucose using a Biosen C_Line automated analyzer (EKF Diagnostics, Cardiff, UK).

Following resting measures, participants then consumed a test breakfast meal (sports drink and porridge) comprising a total carbohydrate load of 2 g·kg^−1^ body mass. This load was based on similar research [32] instigating a fixed 150 g carbohydrate dose, but tailored relative to participant body mass and based on a high-carbohydrate daily plan (~6 g·kg^−1^). The meal consisted of a sports drink (Maurten Hydrogel Sports Fuel Drink Mix 320, Maurten UK Ltd., London, UK; comprising 41 g total carbohydrate per sachet), which was standardized to 75 g of total carbohydrate mixed with 400 mL of water. The remaining carbohydrate load was calculated against individual body mass delivered via a basic porridge meal (Quaker Oats So Simple, Reading, Berkshire, UK; comprising 26 g total carbohydrate per sachet). To standardize fluid intake, the test meal was made with a total of 9 mL·kg^−1^ water (with sports drink water volume deducted). As such, the mean carbohydrate intake for the test meal was 69.1 ± 25.1 g, and mean fluid intake (excluding sports drink) was 248.5 ± 113.0 mL. Mean total carbohydrate load was 144.1 ± 25.1 g. Participants were required to consume the test meal over a 10 min timed period. Further duplicate Lumen and Douglas bag measures and capillarized blood samples were collected post-meal at 30 min (P30) and 60 min (P60) (with the timer started at the beginning of consumption of the test meal).

### Phase 2: main dietary intervention assessment

#### Study participants and eligibility criteria

For the main intervention, sample size power calculation (G*power3, Dusseldorf, Germany [35]) was undertaken using L%CO_2_ data from our laboratory-based study and determined a sample size of 15, using an α = 0.05 and 1 − β = 0.80. Participants were recruited based on the same inclusion criteria as the laboratory-based assessment study. Prior to study inclusion, participants attended a separate study briefing and provided written informed consent. An initial pool of 31 participants took part in the study, from which 4 were eliminated from the final analysis based on non-compliance with the main study protocol (i.e. were either on a hypocaloric diet or did not conform to the study parameters). A cohort of 27 participants (9 males, 18 females) were therefore included in final analyses (Mean ± SE: age: 42 ± 2 yrs; body mass: 71.9 ± 1.9 kg; height: 1.72 ± 0.02 m; BMI: 24.5 ± 0.7 kg·m^2^).

#### Experimental design and intervention

For the main study, a randomized, cross-over, dietary controlled design was implemented. Participants initially attended the laboratory to collect study equipment, comprising a Lumen device (with individual study login/code allocation), a Xiaomi Mi Smart Band 4 heart rate monitor, and a Xiaomi Mi Body Composition Scale 2 (Xiaomi Technology UK Ltd., Reading, UK). During this initial visit, participants were measured for both height (Seca CE123, Hamburg, Germany) and body mass (Seca 780, Hamburg, Germany); with laboratory assessed body mass cross checked against the Xiaomi Mi Smart Scale (mean difference 0.2 ± 0.1 kg, *P* > 0.05). Participants were also provided with guided instructions into the set up and use of the Lumen device, along with an activity diary and individual MyFitnessPal application accounts (MyFitnessPal, Inc., San Francisco, CA, USA).

Participants then had a 10-day habituation period to activate their Lumen application and become familiar with the specific breath maneuver, and gather typical ranges based on minimum/maximum L%CO_2_ measured values. During the final 7-days, participants recorded their normal exercise patterns using the daily activity log provided to account for session type, mean session heart rate, exercise duration, and session perceived exertion. From this mean weekly training load, training monotony and strain were estimated based on previous research [36]. In addition, participants were provided with individual guidance on daily completion of dietary intake using the smart phone application (MyFitnessPal) with due attention to meal content and brands, portion size/weight, and fluid intake, as previously reported by our group [37]. Individual records were assessed daily by the same researcher to ensure satisfactory compliance and completion detail. Food diaries were then assessed by the same researcher using Nutritics Professional Dietary Analysis software (Nutritics Ltd., Co. Dublin, Ireland).

From this initial habituation period (NORM), individual maintenance caloric intake was determined based on predicted basal metabolic rate (using the Harris-Benedict formula), adjusted against habitual training demands and estimated non-exercise activity thermogenesis [38]. Participants were randomly assigned a 7-day dietary intervention comprising either isocaloric low-carbohydrate (LOW) intake (target ratio of ~20–25% EI from carbohydrate, 15–20% protein, 55–60% fat) or isocaloric high-carbohydrate (HIGH) intake (target ratio ~55–60% EI from carbohydrate, 15–20% protein, 20–25% fat). Individual guidance on meal-planning was provided for each dietary intervention. The dietary interventions were interspersed with a ‘wash-out’ period of at least 7 days returning to habitual intake.

Throughout all dietary periods, participants were requested to perform duplicate Lumen device measures at the following daily stipulated periods: i) overnight fasted (~10 h), morning assessment (30 min post-waking) and morning body mass; ii) 45 min post-breakfast; iii) immediately pre-evening meal; iv) 45 min post-evening meal; and v) pre-bed. Instructions were provided for participants to rest in a comfortable seated position for ~5 min before each measured time-point to standardize the process.

#### Quality control

Every Lumen breath session undertaken by the participants was stored through the Lumen application in an online platform, and reviewed by one experienced researcher on the acceptability and repeatability criteria for each breath. Acceptability was based on ambient air %CO_2_ and Lumen breath maneuver requirements for inhaled volume, breath hold time, and exhaled volume. The session %CO_2_ was determined through replicate measures with a difference <0.2%. Breath sessions that did not meet acceptability criteria were removed from the final analysis.

#### Statistical analyses

Statistical analyses were performed using SPSS (v26, IBM, NY, USA). Dependent variable distributions were assessed for normality using a Shapiro-Wilk test, with outlier evaluation using 1.5 x interquartile range. Laboratory-based data were assessed using a one-way ANOVA, with ordinary least squares regression used to assess the model between L%CO_2_ and RER. For the main intervention, a mixed design repeated measures ANOVA (diet, day) was performed for L%CO_2_ and L_I_ score, with Bonferroni post-hoc assessment where applicable. Where sphericity was violated, a Greenhouse–Geisser correction was applied. An α level of ≤0.05 was employed for statistical significance, with effect size (partial eta squared; η_p_^2^) also reported (small = 0.02, medium = 0.13, large = 0.26). Data are reported as the mean ± SE.

## Results

### Phase 1: laboratory-based device assessment

**Figure 1.**
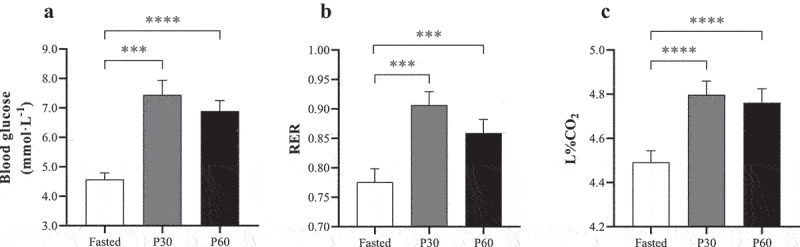
Comparison between fasted and post-meal (30 and 60 min) laboratory and Lumen device measures: (a) blood glucose (mmol·L^−1^); (b) expired air respiratory exchange ratio (RER); and (c) Lumen%CO_2_ (L%CO_2_). *** Denotes significant difference between paired timepoints (*P* ≤ 0.003). **** Denotes significant difference between paired timepoints (*P* < 0.001).

Laboratory-based assessment of the Lumen device is shown in Figure 1 for mean blood glucose, RER, and L%CO_2_. Blood glucose significantly increased (F = 21.82, *P* < 0.001, η_p_^2^ = 0.67) from 4.57 ± 0.22 to a peak of 7.44 ± 0.49 mmol·L^−1^ by 30 min post-meal (*P* = 0.001), and remained elevated at 60 min (6.89 ± 0.36 mmol·L^−1^; *P* < 0.001 compared with rest). Likewise, RER peaked at 30 min post-meal (F = 16.37, *P* < 0.001, η_p_^2^ = 0.62), increasing by 18.1% to 0.91 ± 0.02 (*P* = 0.002) and remained elevated compared with resting RER at 60 min (0.86 ± 0.02; *P* = 0.003). The impact of the test meal also resulted in a significant increase in L%CO_2_ (F = 30.49, *P* < 0.001, η_p_^2^ = 0.74) from 4.49 ± 0.05% to 4.80 ± 0.06% by 30 min, remaining elevated at 4.76 ± 0.06% at 60 min (*P* < 0.001). For all measures, no differences were observed between P30 and P60 (*P* > 0.05).

Mean Douglas bag data are shown in Table 1. No significant differences were reported over time for fractional expired oxygen percentage (F_E_O_2_%) or VO_2_ (L·min^−1^; *P* > 0.05). Fractional expired %CO_2_ (F_E_CO_2_%) increased significantly over time (F = 4.48, *P* = 0.03, η_p_^2^ = 0.31), with post-hoc analysis demonstrating a significant increase by P30 (*P* = 0.047) but not P60 (*P* > 0.05). However, VCO_2_ (L·min^−1^) significantly increased over time (F = 15.90, *P* < 0.001, η_p_^2^ = 0.61) both at P30 (*P* = 0.005) and P60 (*P* = 0.001) compared with fasted. Minute ventilation (V_E_) also increased over time (F = 8.42, *P* = 0.002, η_p_^2^ = 0.46), with paired significance only demonstrated at P60 compared with fasted (*P* = 0.014).

**Table 1.** Mean Douglas bag data for fasted and post-meal respiratory measures.

Variable	Fasted	P30	P60
F_E_O_2_ (%)	18.27 ± 0.20	18.44 ± 0.19	18.39 ± 0.18
F_E_CO_2_ (%)	2.13 ± 0.12	2.30 ± 0.14*	2.26 ± 0.12
V_E_ (L·min^−1^)	13.62 ± 1.36	15.48 ± 1.31	17.19 ± 1.35*
VO_2_ (L·min^−1^)	0.36 ± 0.03	0.38 ± 0.02	0.42 ± 0.02
VCO_2_ (L·min^−1^)	0.28 ± 0.02	0.34 ± 0.02*	0.37 ± 0.02*
RER	0.77 ± 0.03	0.91 ± 0.02*	0.86 ± 0.02*

Data presented as mean ± SE. F_E_O_2_ (%) = fraction of expired oxygen (%); F_E_CO_2_ (%) = fraction of expired carbon dioxide (%); V_E_ = absolute minute ventilation (STPD); VO_2_ = absolute oxygen consumption standardized; VCO_2_ (STPD) = absolute carbon dioxide standardized (STPD); RER = respiratory exchange ratio. * = significantly different to fasted (*P* ≤ 0.047).

**Figure 2.**
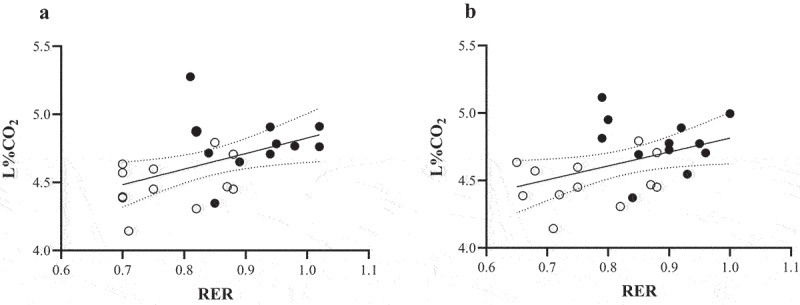
Ordinary least squares regression between expired RER and Lumen %CO_2_ (L%CO_2_) for: (a) fasting and 30 min post-meal (P30); (b) fasting and mean post-meal data. Open circles indicate fasting measures; closed circles represent respective fed measures.

Ordinary least squares regression was employed to assess the model fit between RER and L%CO_2_ using fasted and post-meal measures. When considering peak (P30) values, a significant regression model was found (F = 5.62, *P* = 0.03, R^2^ = 0.20; see Figure 2 tile A) with L%CO_2_ found to significantly predict RER (β = 1.042, *P* = 0.03). When mean post-meal data were considered, a significant model effect was also similarly found (F = 5.35, *P* = 0.03, R^2^ = 0.20; see Figure 2 tile B). Based on these models, it was estimated that each 0.1 unit increase in RER corresponded with an 0.11% increase in L%CO_2_.

### Phase 2: main dietary intervention assessment

#### Dietary monitoring and training load

Dietary intake adherence to the intervention is shown in Table 2. No significant differences were observed for absolute or relative caloric intake between LOW or HIGH, or compared with prior habitual intake (*P* > 0.05). Furthermore, no differences were observed between estimated maintenance kilocalories (2277.1 ± 60.3 kcal·d^−1^) compared with caloric intake during both LOW and HIGH (*P* > 0.05). Protein intake as a percentage of caloric intake was equally maintained across both LOW (19.9 ± 0.3%) and HIGH (19.3 ± 0.4%) interventions (*P* > 0.05), in accordance with the targeted maintenance kilocalories.

**Table 2.** Mean dietary intake at baseline and across intervention periods.

		NORM	LOW	HIGH
EI	(kcal·d^−1^)	2196.3 ± 77.2	2270.9 ± 65.8	2236.3 ± 72.4
	(kcal·kg^−1^·d^−1^)	31.0 ± 1.3	32.2 ± 1.1	31.3 ± 1.3
CHO	(%EI)	41.7 ± 1.9 *	19.9 ± 0.6	56.3 ± 0.8 ^#^
	(g·d^−1^)	231.3 ± 14.4 *	113.2 ± 4.9	315.4 ± 12.3 ^#^
	(g·kg^−1^·d^−1^)	3.3 ± 0.2 *	1.6 ± 0.1	4.4 ± 0.2 ^#^
PRO	(%EI)	18.0 ± 0.6 ^a^	19.9 ± 0.3	19.3 ± 0.4
	(g·d^−1^)	98.1 ± 4.0 ^b^	113.2 ± 3.6	107.9 ± 3.9
	(g·kg^−1^·d^−1^)	1.4 ± 0.1 ^b^	1.6 ± 0.1	1.5 ± 0.1
FAT	(%EI)	36.9 ± 1.6 *	57.7 ± 0.8	22.8 ± 0.6 ^#^
	(g·d^−1^)	89.4 ± 4.5 *	145.4 ± 4.3	56.2 ± 1.9 ^#^
	(g·kg^−1^·d^−1^)	1.3 ± 0.1 *	2.1 ± 0.1	0.8 ± 0.0 ^#^

Data presented as mean ± SE. NORM = normal dietary intake pre-intervention; LOW = low carbohydrate diet week; HIGH = high carbohydrate diet week. EI = caloric energy intake; CHO = carbohydrate; PRO = protein. * = significantly different to both LOW and HIGH (*P* < 0.001); ^#^ = significantly different to LOW (*P* < 0.001); ^a^ = significantly different to LOW only (*P* = 0.037); ^b^ = significantly different to both LOW and HIGH (*P* ≤ 0.03).

As expected, a significant main effect was found for total carbohydrate intake (F = 149.23, *P* < 0.001, η_p_^2^ = 0.85). For LOW, mean carbohydrate intake was at the lower range of the targeted 20–25% EI (19.9 ± 0.6%) and was significantly lower than both NORM and HIGH (*P* < 0.001). For HIGH, mean carbohydrate intake satisfactorily met the targeted 55–60% EI range (56.3 ± 0.8%) and was significantly greater than both NORM and LOW demonstrating adherence to the dietary interventions overall (*P* < 0.001). Likewise, there was a significant main effect for total fat intake (F = 258.54, *P* < 0.001, η_p_^2^ = 0.91), with mean relative contribution satisfactorily meeting the targeted 55–60% EI for LOW (57.7 ± 0.8%) and 20–25% EI for HIGH (22.8 ± 0.6%), in both cases being significantly different to NORM and the opposite intervention (*P* < 0.001). No significant differences were reported for body mass at the start of each dietary period (NORM: 71.9 ± 1.9 kg; LOW: 71.6 ± 2.0 kg; HIGH: 72.7 ± 2.3 kg, *P* > 0.05). Training load comparisons across all dietary periods are shown in Table 3. No significant differences were reported between dietary periods for any of the training load variables (*P* > 0.05) indicating relative consistency across the study duration.

**Table 3.** Training load comparisons across dietary periods.

Variable	NORM	LOW	HIGH
sTIME (mins)	54.0 ± 2.1	58.9 ± 2.3	58.7 ± 3.3
sHR (b·min^−1^)	133 ± 3	133 ± 4	132 ± 4
sRPE (0-10)	5.3 ± 0.2	5.4 ± 0.2	5.5 ± 0.2
TL (AU)	1581.4 ± 125.1	1666.0 ± 146.5	1676.9 ± 129.6
TM (AU)	1.3 ± 0.1	1.2 ± 0.1	1.2 ± 0.1
TS (AU)	1863.5 ± 227.3	1784.8 ± 211.3	1881.1 ± 218.4

Data presented as mean ± SE. sTIME = mean exercise session duration; sHR = mean session heart rate; sRPE = mean session perceived exertion rating using a 0–10 visual analogue scale; TL = mean weekly intervention training load (AU; arbitrary units); TM = mean weekly training monotony based on TL; TS = mean weekly estimated training strain.

#### Lumen response to dietary interventions

**Figure 3.**
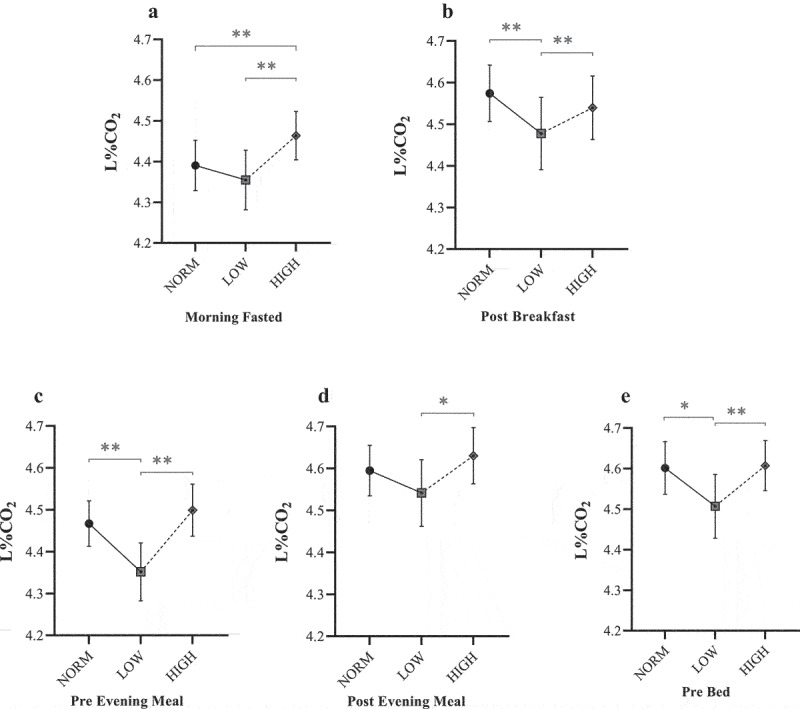
Mean Lumen %CO_2_ responses for NORM, LOW, and HIGH according to: (a) ~30 min post-waking fasted assessment; (b) ~45 min post-breakfast; (c) immediately before evening meal; (d) ~45 min post evening meal; and (e) immediately before bed. * Denotes significant difference between paired timepoints (*P* ≤ 0.043). ** Denotes significant difference between paired timepoints (*P* ≤ 0.009).

**Figure 4.**
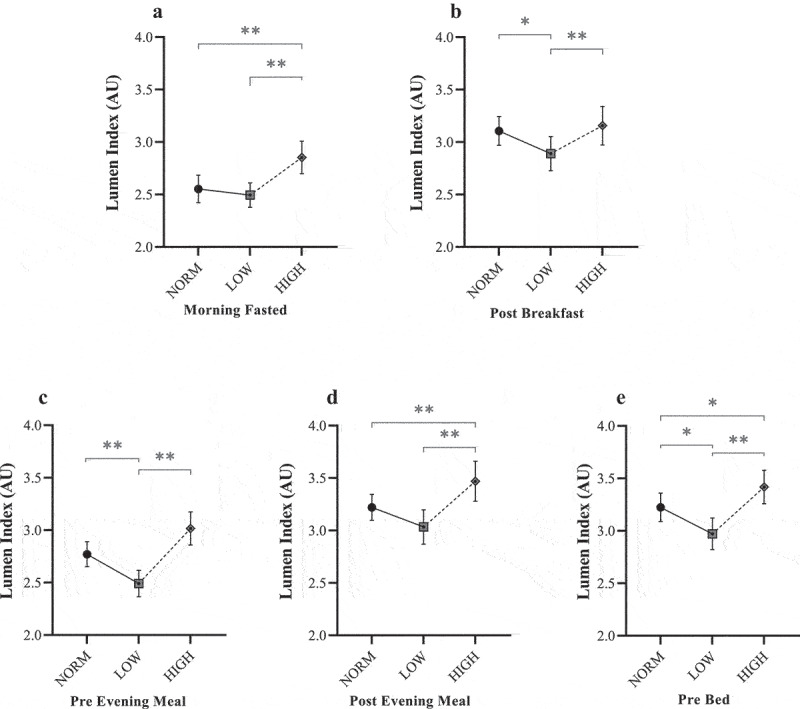
Mean Lumen Index responses for NORM, LOW, and HIGH according to: (a) ~30 min post-waking fasted assessment; (b) ~45 min post-breakfast; (c) immediately before evening meal; (d) ~45 min post evening meal; and (e) immediately before bed. AU = arbitrary units. * Denotes significant difference between paired timepoints (*P* ≤ 0.028). ** Denotes significant difference between paired timepoints (*P* ≤ 0.005).

Mean L%CO_2_ and L_I_ values across each dietary period are shown in Figures 3 and 4, respectively. Under fasted conditions, there was a significant main diet effect for L%CO_2_ (F = 8.05, *P* < 0.001, η_p_^2^ = 0.05) and L_I_ (F = 10.84, *P* < 0.001, η_p_^2^ = 0.07). For HIGH, mean fasted L%CO_2_ (4.46 ± 0.06%) and L_I_ (2.9 ± 0.2 arbitrary units [AU]) were significantly greater than both NORM (*P* < 0.005) and LOW (*P* < 0.001). However, no post-hoc differences were observed between NORM and LOW (*P* > 0.05).

There was also a main diet effect found for L%CO_2_ (F = 7.30, *P* < 0.001, η_p_^2^ = 0.04) and L_I_ (F = 7.49, *P* < 0.001, η_p_^2^ = 0.04) when considering mean values over each dietary period in relation to the first meal of the day. In contrast to morning fasted values, post-hoc analysis revealed significantly lower values following LOW (L%CO_2_ = 4.48 ± 0.09%, L_I_ = 2.9 ± 0.2 AU) compared with both NORM (L%CO_2_ = 4.57 ± 0.07%, L_I_ = 3.1 ± 0.1 AU: *P* < 0.023) and HIGH (L%CO_2_ = 4.54 ± 0.08%, L_I_ = 3.2 ± 0.2 AU; *P* < 0.004).

Lumen values typically decreased by the evening meal. For the pre-evening meal measures, a significant main effect was found for both L%CO_2_ (F = 19.59, *P* < 0.001, η_p_^2^ = 0.10) and L_I_ (F = 19.12, *P* < 0.001, η_p_^2^ = 0.10), demonstrating a similar pattern to post-breakfast measures. Post-hoc analyses demonstrated a significantly lower L%CO_2_ (4.35 ± 0.07%) and L_I_ (2.5 ± 0.1 AU) for LOW compared with both NORM and HIGH (*P* < 0.001), although no differences were reported between NORM and HIGH (*P* > 0.05). The consensus between L%CO_2_ and L_I_ was consistent until post-dinner measures. At this point, there was a main effect for L%CO_2_ (F = 5.87, *P* = 0.005, η_p_^2^ = 0.03), whereby mean L%CO_2_ was significantly different between intervention diets only (LOW = 4.54 ± 0.08%; HIGH = 4.63 ± 0.07%; *P* = 0.01). Whilst this same comparison was also detected for L_I_ (F = 12.13, *P* < 0.001, η_p_^2^ = 0.06), values were considerably greater for HIGH (3.5 ± 0.2 AU) compared to both LOW (3.0 ± 0.2 AU; *P* < 0.001) and NORM (3.2 ± 0.1 AU; *P* = 0.005). Post-dinner measures were not different between NORM and LOW (*P* > 0.05).

For the final measure for each day (pre-bed), a significant main effect was found for both L%CO_2_ (F = 6.18, *P* = 0.003, η_p_^2^ = 0.03) and L_I_ (F = 14.97, *P* < 0.001, η_p_^2^ = 0.08). For L%CO_2_, values were notably lower following LOW (4.51 ± 0.08%) compared with both NORM (4.60 ± 0.06%; *P* = 0.04) and HIGH (4.61 ± 0.06%; *P* = 0.005), respectively. Similarly, following LOW, L_I_ values were significantly lower (3.0 ± 0.2 AU) than both NORM (3.2 ± 0.1 AU; *P* = 0.02) and HIGH (3.4 ± 0.2 AU; *P* < 0.001). However, it was also noted that a significant difference existed between NORM and HIGH (*P* = 0.03) in contrast to the pattern for L%CO_2_.

## Discussion

The aim of the current study was to undertake an independent assessment of the efficacy of the Lumen breath device to track %CO_2_ in response to acute dietary changes in healthy volunteers under real-world conditions. As part of this, we also undertook an independent assessment of the Lumen device under controlled conditions. Findings from our laboratory assessment concurred with those previously reported [32], demonstrating that glycemic response to a carbohydrate-rich test-meal corresponds with distinctive increases in both RER and L%CO_2_. In the current study, however, we employed a fixed meal plan in contrast to previous research [32] which provided three 50 g glucose solutions separated by 20 min each. This was to instigate a more realistic feeding strategy in line with a high-carbohydrate meal approach. In the current study using the Douglas bag method, an 18.1% (or 0.14 unit) increase in RER corresponded with a 6.9% increase in L%CO_2_ (or 0.31 absolute %CO_2_ increase). Lorenz et al. [32] similarly demonstrated an 11.0% increase in RER (or 0.09 unit), using a metabolic cart, corresponded with a 6.6% increase in L%CO_2_ (or 0.28%CO_2_ increase). We also demonstrated a significant regression model effect between RER and L%CO_2_ using both peak (P30) values (*P* = 0.03, R^2^ = 0.20) and mean post-fed values (*P* = 0.03, R^2^ = 0.20), again comparable to previous research [32]. Expired air analysis demonstrated that changes in RER following the carbohydrate meal likely reflect an increased VCO_2_ (~940 mL), with a smaller (~673 mL) non-significant change in VO_2_.

Our findings are consistent with previous research [39–46], whereby post-prandial VCO_2_ production is associated with increased V_E_ to facilitate constant arterial CO_2_ tension (PaCO_2_). This also highlights the critical importance of standardizing resting procedures as part of Lumen measurements to ensure stable O_2_ use. Our results therefore infer that relative changes in L%CO_2_ in response to a test meal may be useful for tracking corresponding metabolic changes based on acute shifts in CO_2_ production, on the assumption that O_2_ consumption remains relatively constant. As the Lumen device does not measure %O_2_, more accurate quantification of metabolic changes cannot be ascertained. Instead, the device may be pertinent for indirect tracking of metabolic alterations to carbohydrate-based meal patterns, and further validation work is warranted.

For the main study, dietary consistency was generally met across the intervention stages, with mean macronutrient distribution ranges achieved for both LOW and HIGH carbohydrate weeks. An important finding from our study was that when macronutrient ratios (particularly for carbohydrate intake) were significantly polarized (i.e. ~20% vs. 60% of caloric load), consistent and significant differences were found for both mean L%CO_2_ and L_I_ for all timepoints assessed. However, no interactions between diet and time were found for any measures, indicating that day-to-day changes were not significant. These findings therefore highlight that mean weekly changes in L%CO_2_ and L_I_ measures may provide better indicators of general metabolic adaptations in response to acute dietary changes, particularly when the macronutrient ‘shifts’ are significant. This may have important implications for end-user interpretations when using the device.

One contention with this suggestion, however, is that end-users do not view real-time L%CO_2_ data, and instead only view a derived L_I_ to provide an indicator of metabolic fuel contribution (based on regression agreement between L%CO_2_ and RER). However, a critical insight is that this largely depends on the spread of variance in L%CO_2_ values and, indeed, assumed RER range for a particular individual. From a metabolic perspective, whilst a low L_I_ score (i.e. 1) may be associated with a lower relative RER value, if the distribution of L%CO_2_ is narrow, then presumed metabolic changes may be minimal. Additionally, RER values typically range from 0.7 to 1.0 under resting conditions, with extremes of this range associated with fat and carbohydrate utilization respectively (and mixed fuel utilization in between). This has important implications for end-user interpretation of results, particularly if assessing acute changes or if resting procedures are not appropriately standardized.

Furthermore, in the current study, mean L_I_ data were compiled to one decimal place to reflect the subtle changes observed. Indeed, across both dietary interventions, mean L_I_ ranged from 2.5 (morning fasted on LOW) to 3.5 (post evening meal on HIGH), supporting the small effect sizes observed. Whilst it was expected that the transition to a low-carbohydrate diet might result in a consecutive reduction in L%CO_2_ and L_I_ under fasting conditions as example, this was only partly observed over days 2–4, after which measures fluctuated in line with potential metabolic adaptations (i.e. ketosis [47,48]) resulting in subtle increases in L%CO_2_. As such, end-users should be mindful of tracking mean weekly L_I_ to have a more reliable understanding of individual responses to acute dietary patterns at a particular point in the day.

When comparing differences between NORM and LOW, our findings indicated that statistically significant responses occurred for both mean L%CO_2_ and L_I_ post-breakfast, pre-evening meal, and pre-bed. However, differences were not found under fasting conditions. This may indicate that metabolic adaptations to an acute low-carbohydrate diet are better observed at these times, with lack of significance under fasting conditions potentially reflecting acute ketogenic influence on CO_2_ fluctuations in the latter half of the week. Longer term dietary monitoring is therefore warranted to clarify these findings, especially in light of ketogenic adaptations to prolonged low-carbohydrate or intermittent fasting protocols [49,50].

In contrast, the main time point where significant differences were observed between NORM and HIGH for both mean L%CO_2_ and L_I_ was under fasting conditions. Mean carbohydrate intake under NORM represented 41.7% of EI compared with 56.3% for HIGH. Increased consumption of carbohydrate over the intervention period may have influenced carbohydrate oxidation rates [51,52] leading to elevated expired CO_2_ under fasted conditions. Potentially, this infers that when dietary carbohydrate is significantly increased, Lumen measures tracked under fasting conditions may offer a more reliable or standardized time-point. However, further research is required to corroborate these observations.

It is important to recognize several limitations of the current study. Whilst sample size was deemed sufficient, a larger sample pool representative of wider consumer, athletic, or clinical populations is warranted to determine the effectiveness of the Lumen device in different settings. Indeed, the current study involved recreationally active volunteers, and expired percentage CO_2_ may be elevated in trained athletes [53]. Furthermore, circadian hormonal patterns (i.e. across the menstrual cycle) were not determined which may influence metabolic responses to dietary interventions. Further research on the efficacy of the Lumen device, particularly with female users, is therefore warranted to determine endocrine influence on L%CO_2_ variance. As this study aimed to assess device efficacy under real-world settings, specific meal volume/quantity and set timings were not controlled for. In the current study, overall effect sizes were relatively small; therefore, when strict meal type/amount and timings are followed, metabolic responses based on expired CO_2_ may be greater. Furthermore, whilst overnight fasting periods were appropriately standardized (~10 h), longer term fasting periods may yield different results.

Whilst the current study demonstrates that the Lumen device may provide useful mean weekly differences between polarized dietary conditions, day-to-day variance did not yield significant findings. Therefore, future research should aim to provide further confirmation on the efficacy and accuracy of the device under controlled laboratory conditions (i.e. with standardized test meals), or with progressive increments of carbohydrate intake to determine whether a ‘threshold’ level for meaningful detection exists. As the current study investigated acute dietary interventions, it would also be meaningful to determine longer term patterns with device use when undertaking dietary programs (e.g. ketogenic diets) alongside laboratory (e.g. RER, blood ketone assessment) or other proxy measures (e.g. glucose monitoring, breath acetone).

### Translational aspects

Our findings support previous research [32] that the device detects acute changes in L%CO_2_ following a significant increase in carbohydrate intake, and collectively these results demonstrate that the Lumen device could have pertinent applications in non-invasively monitoring response to acute meal settings (e.g. pre-exercise carbohydrate strategies) considering previous research highlighting the association between carbohydrate oxidation and exercise performance [18,38,54–56].

For the main aspect of this study, our findings also highlight that the device could be useful for monitoring mean weekly changes in L%CO_2_ and L_I_ in response to acute dietary approaches (i.e. carbohydrate reduction for weight loss, or carbohydrate-loading for endurance events). However, our results did not demonstrate day-to-day changes at a particular time-point, which may have pertinent applications for end-users when interpreting acute differences between days. The Lumen device may therefore be pertinent for monitoring relative consistency to a targeted nutrition program, with applications to research and individual-user contexts (e.g. at-home monitoring of adherence to a low- or high-carbohydrate diet).

It is important to note that results may be different for chronic interventions (particularly in line with assessment of metabolic flexibility) and further studies should assess the efficacy and practical benefits of using the device longitudinally. End-users should be mindful that metabolic responses to dietary interventions are both complex and highly individualized. This is further apparent when considering differences between trained/lean and untrained/obese populations. Whilst dietary interventions (e.g. low-carbohydrate ketogenic, fat restricted or targeted energy-reduced diets) may modify substrate utilization (i.e. an increase in fat oxidation) leading to improved body composition [57,58], particularly in overweight individuals [47,59], this may not necessarily confer performance benefits (e.g. increased peak power or time to exhaustion [57,60], prolonged endurance [58], or efficient glycogen use [61]). Use of additional measures (e.g. body composition, performance data) may therefore be relevant as an adjunct strategy to support application of the Lumen device. Finally, further research should be undertaken to assess device efficacy in relation to specific end-user goals pertinent to metabolic outcomes [62].

## Conclusion

A portable, home-use metabolic device (Lumen) detected significantly increased expired %CO_2_ in response to a high-carbohydrate test meal, and may be useful in tracking mean weekly changes to acute dietary carbohydrate modifications, especially when protein intake is standardized. Lumen measures following a high-carbohydrate diet may be better assessed under fasting conditions, whereas measures post-breakfast may be better suited for monitoring low-carbohydrate diets. Further research comparing applied versus laboratory settings is warranted to establish clinical and practical efficacy of the Lumen device, particularly in relation to end-user goals.

## Abbreviations

BMI: body mass index; EI: energy intake; F_E_CO_2_: fraction of expired carbon dioxide; F_E_O_2_: fraction of expired oxygen; HIGH: high-carbohydrate diet intervention; L%CO_2_: Lumen breath percent carbon dioxide; L_I_: Lumen Index; LOW: low-carbohydrate diet intervention; NORM: habitual diet intake; RER: respiratory exchange ratio; RQ: respiratory quotient; VCO_2_: volume of expired carbon dioxide; VO_2_: volume of oxygen consumption; V_E_: minute ventilation.

## Data sharing statement

The data collected and analyzed in this study are available from the corresponding author upon reasonable request (https://aru.ac.uk/people/justin-roberts).

## References

[1] Jeukendrup AE. Carbohydrate intake during exercise and performance. Nutrition. 2004;20:669–86. 15212750 10.1016/j.nut.2004.04.017

[2] Stellingwerff T, Morton JP, Burke LM. A framework for periodized nutrition for athletics. Int J Sport Nutr Exerc Metab. 2019;29(2):141–151. 30632439 10.1123/ijsnem.2018-0305

[3] Jeukendrup AE. Periodized nutrition for athletes. Sports Med. 2017;47(Suppl 1):51–63. 28332115 10.1007/s40279-017-0694-2 PMC5371625

[4] Cox GR, Clark SA, Cox AJ, et al. Daily training with high carbohydrate availability increases exogenous carbohydrate oxidation during endurance cycling. J Appl Physiol. 2010;109:126–134. 20466803 10.1152/japplphysiol.00950.2009

[5] Antonio J, Candow DG, Forbes SC, et al. Effects of dietary protein on body composition in exercising individuals. Nutrients. 2020;12(6):1890. 32630466 10.3390/nu12061890 PMC7353221

[6] Zoorob R, Parrish ME, O’Hara H, et al. Sports nutrition needs: before, during, and after exercise. Prim Care. 2013;40(2):475–486. 23668654 10.1016/j.pop.2013.02.013

[7] Thomas DT, Erdman KA, Burke LM. Position of the academy of nutrition and dietetics, dietitians of Canada, and the American College of Sports Medicine: nutrition and athletic performance. J Acad Nutr Diet. 2016;116(3):501–528. 26920240 10.1016/j.jand.2015.12.006

[8] Spriet LL, Peters SJ. Influence of diet on the metabolic responses to exercise. Proc Nutr Soc. 1998;57(1):25–33. 9571705 10.1079/pns19980006

[9] Shephard RJ. Open-circuit respirometry: a brief historical review of the use of Douglas bags and chemical analyzers. Eur J Appl Physiol. 2017;117:381–387. 28210818 10.1007/s00421-017-3556-6

[10] Mtaweh H, Tuira L, Floh AA, et al. Indirect calorimetry: history, technology, and application. Front Pediatr. 2018;6:257. 30283765 10.3389/fped.2018.00257 PMC6157446

[11] Goedecke JH, Clair Gibson A, Grobler L, et al. Determinants of the variability in respiratory exchange ratio at rest and during exercise in trained athletes. Am J Physiol Endocrinol Metab. 2000;279(6):E1325–E1334. 11093921 10.1152/ajpendo.2000.279.6.E1325

[12] Macfarlane DJ. Open-circuit respirometry: a historical review of portable gas analysis systems. Eur J Appl Physiol. 2017;117:2369–2386. 29043499 10.1007/s00421-017-3716-8

[13] Ross R, ALDuhishy A, González-Haro C. Validation of the cosmed K4b2 portable metabolic system during running outdoors. J Strength Cond Res. 2020;34(1):124–133. 30707139 10.1519/JSC.0000000000003050

[14] Roffey DM, Byrne NM, Hills AP. Day-to-day variance in measurement of resting metabolic rate using ventilated-hood and mouthpiece & nose-clip indirect calorimetry systems. JPEN J Parenter Enteral Nutr. 2006;30(5):426–432. 16931612 10.1177/0148607106030005426

[15] Jeukendrup AE, Wallis GA. Measurement of substrate oxidation during exercise by means of gas exchange measurements. Int J Sports Med. 2005;26(1):28–37. 10.1055/s-2004-830512 15702454

[16] Hulston CJ, Wallis GA, Jeukendrup AE. Exogenous CHO oxidation with glucose plus fructose intake during exercise. Med Sci Sports Exerc. 2009;41(2):357–363. 19127189 10.1249/MSS.0b013e3181857ee6

[17] Jentjens RLPG, Jeukendrup AE. High rates of exogenous carbohydrate oxidation from a mixture of glucose and fructose ingested during prolonged cycling exercise. Br J Nutr. 2005;93(4):485–492. 15946410 10.1079/bjn20041368

[18] Roberts JD, Tarpey MD, Kass LS, et al. Assessing a commercially available sports drink on exogenous carbohydrate oxidation, fluid delivery and sustained exercise performance. J Int Soc Sports Nutr. 2014;11(1):8. 24589205 10.1186/1550-2783-11-8 PMC3975841

[19] Tarpey MD, Roberts JD, Kass LS, et al. The ingestion of protein with a maltodextrin and fructose beverage on substrate utilisation and exercise performance. Appl Physiol Nutr Metab. 2013;38(12):1245–1253. 24195625 10.1139/apnm-2012-0306

[20] González-Haro C. Concordance between 13 C:12 C ratio technique respect to indirect calorimetry to estimate carbohydrate and fat oxidation rates by means stoichiometric equations during exercise. A reliability and agreement study. Physiol Rep. 2019;7(8):e14053. 31025485 10.14814/phy2.14053 PMC6483938

[21] Rosenkilde M, Nordby P, Nielsen LB, et al. Fat oxidation at rest predicts peak fat oxidation during exercise and metabolic phenotype in overweight men. Int J Obes (Lond). 2010;34(5):871–877. 20157319 10.1038/ijo.2010.11

[22] Rynders CA, Blanc S, DeJong N, et al. Sedentary behaviour is a key determinant of metabolic inflexibility. J Physiol. 2018;596(8):1319–1330. 28543022 10.1113/JP273282 PMC5899985

[23] Waldman HS, Bryant AR, Knight SN, et al. Assessment of metabolic flexibility by substrate oxidation responses and blood lactate in women expressing varying levels of aerobic fitness and body fat. J Strength Cond Res. 2023;37(3):581–588. 10.1519/JSC.0000000000004316 35836305

[24] San-Millán I, Brooks GA. Assessment of metabolic flexibility by means of measuring blood lactate, fat, and carbohydrate oxidation responses to exercise in professional endurance athletes and less-fit individuals. Sports Med. 2018;48(2):467–479. 28623613 10.1007/s40279-017-0751-x

[25] Zdzieblik D, Friesenborg H, Gollhofer A, et al. Effect of a high fat diet vs. high carbohydrate diets with different glycemic indices on metabolic parameters in male endurance athletes: a pilot trial. Front Nutr. 2022;9:802374. 35479739 10.3389/fnut.2022.802374 PMC9037589

[26] Seo HC, Shin D, Leem CH, et al. Development of a portable respiratory gas analyzer for measuring indirect resting energy expenditure (REE). J Healthc Eng. 2021;2021:8870749. 33680417 10.1155/2021/8870749 PMC7904359

[27] Guidetti L, Meucci M, Bolletta F, et al. Validity, reliability and minimum detectable change of COSMED K5 portable gas exchange system in breath-by-breath mode. PLoS One. 2018;13(12):e0209925. 30596748 10.1371/journal.pone.0209925 PMC6312326

[28] Tsekouras YE, Tambalis KD, Sarras SE, et al. Validity and reliability of the new portable metabolic analyzer PNOE. Front Sports Act Living. 2019;1:24. 33344948 10.3389/fspor.2019.00024 PMC7739780

[29] Rodbard D. Continuous glucose monitoring: a review of successes, challenges, and opportunities. Diabetes Technol Ther. 2016;18(Suppl2):S3–S13. 10.1089/dia.2015.0417 PMC4717493 26784127

[30] Ramos-Jiménez A, Hernández-Torres RP, Torres-Durán PV, et al. The respiratory exchange ratio is associated with fitness indicators both in trained and untrained men: a possible application for people with reduced exercise tolerance. Clin Med Circ Respirat Pulm Med. 2008;2:1–9. 10.4137/ccrpm.s449 PMC2990231 21157516

[31] Melzer K. Carbohydrate and fat utilization during rest and physical activity. e-SPEN. 2011;6(2):e45–e52.

[32] Lorenz KA, Yeshurun S, Aziz R, et al. A handheld metabolic device (Lumen) to measure fuel utilization in healthy young adults: device validation study. Interact J Med Res. 2021;10(2):e25371. 33870899 10.2196/25371 PMC8167606

[33] Wijngaarden MA, Bakker LE, van der Zon GC, et al. Regulation of skeletal muscle energy/nutrient-sensing pathways during metabolic adaptation to fasting in healthy humans. Am J Physiol Endocrinol Metab. 2014;307(10):E885–E895. 25249505 10.1152/ajpendo.00215.2014

[34] Terink R, Witkamp RF, Hopman MT, et al. A 2 week cross-over intervention with a low carbohydrate, high fat diet compared to a high carbohydrate diet attenuates exercise-induced cortisol response, but not the reduction of exercise capacity, in recreational athletes. Nutrients. 2021;13(1):157. 33418951 10.3390/nu13010157 PMC7825040

[35] Faul F, Erdfelder E, Lang A-G, et al. G*power 3: a flexible statistical power analysis program for the social, behavioral, and biomedical sciences. Behav Res Methods. 2007;39:175–191. 17695343 10.3758/bf03193146

[36] Roberts JD, Willmott AGB, Beasley L, et al. The impact of decaffeinated green tea extract on fat oxidation, body composition and cardio-metabolic health in overweight, recreationally active individuals. Nutrients. 2021;13(3):764. 33652910 10.3390/nu13030764 PMC7996723

[37] Roberts J, Zinchenko A, Mahbubani KT, et al. Satiating effect of high protein diets on resistance-trained individuals in energy deficit. Nutrients. 2018;11(1):56. 30597865 10.3390/nu11010056 PMC6356668

[38] Furber M, Pyle S, Roberts M, et al. Comparing acute, high dietary protein and carbohydrate intake on transcriptional biomarkers, fuel utilisation and exercise performance in trained male runners. Nutrients. 2021;13(12):4391. 34959943 10.3390/nu13124391 PMC8706924

[39] Laaban JP, Lemaire F, Baron JF, et al. Influence of caloric intake on the respiratory mode during mandatory minute volume ventilation. Chest. 1985;87:67–72. 3917394 10.1378/chest.87.1.67

[40] Bessard T, Schutz Y, Jequier E. Energy expenditure and postprandial thermogenesis in obese women before and after weight loss. Am J Clin Nutr. 1983;38:680–693. 6637860 10.1093/ajcn/38.5.680

[41] Kelly KP, McGuinnes OP, Buchowski M, et al. Eating breakfast and avoiding late-evening snacking sustains lipid oxidation. PLoS Biol. 2020;18(2):e3000622. 32108181 10.1371/journal.pbio.3000622 PMC7046182

[42] Hayashi K, Suekuni M, Sugiyama K. Effect of food intake on respiratory chemosensitivity to CO2 in young adults. J Physiol Anthro. 2019;38:8. 10.1186/s40101-019-0200-7 PMC6615250 31287028

[43] Whitley HA, Humphreys SM, Campbell IT, et al. Metabolic and performance responses during endurance exercise after high-fat and high-carbohydrate meals. J Appl Physiol. 1998;85(2):418–424. 9688714 10.1152/jappl.1998.85.2.418

[44] Habas ME, Macdonald IA. Metabolic and cardiovascular responses to liquid and solid test meals. Br J Nutr. 1998;79:241–247. 9577302 10.1079/bjn19980041

[45] Efthimiou J, Mounsey PJ, Benson DN, et al. Effect of carbohydrate rich versus fat loads on gas exchange and walking performance in patients with chronic obstructive lung disease. Thorax. 1992;47:451–456. 1496505 10.1136/thx.47.6.451 PMC463811

[46] Gregory S, Wood R, Matthews T, et al. Substrate utilization is influenced by acute dietary carbohydrate intake in active, healthy females. J Sports Sci Med. 2011;10:59–65. 24149296 PMC3737902

[47] Hall KD, Bemis T, Brychta R, et al. Calorie for calorie, dietary fat restriction results in more body fat loss than carbohydrate restriction in people with obesity. Cell Metab. 2015;22(3):427–436. 26278052 10.1016/j.cmet.2015.07.021 PMC4603544

[48] Fried PI, McClean PA, Phillipson EA, et al. Effect of ketosis on respiratory sensitivity to carbon dioxide in obesity. N Engl J Med. 1976;294:1081–1086. 1256524 10.1056/NEJM197605132942003

[49] Ravussin R, Beyl RA, Poggiogalle E, et al. Early time-restricted feeding reduces appetite and increases fat oxidation but does not affect energy expenditure in humans. Obesity. 2019;27:1244–1254. 31339000 10.1002/oby.22518 PMC6658129

[50] Rubini A, Bosco G, Lodi A, et al. Effects of twenty days of the ketogenic diet on metabolic and respiratory parameters in healthy subjects. Lung. 2015;193(6):939–945. 26410589 10.1007/s00408-015-9806-7

[51] Backhouse SH, Williams C, Stevenson E, et al. Effects of the glycemic index of breakfast on metabolic responses to brisk walking in females. Eur J Clin Nutr. 2007;61:590–596. 17136034 10.1038/sj.ejcn.1602566

[52] Podlogar T, Debevec T. Effects of a 14-day high carbohydrate diet on exercise performance of a low-carbohydrate adapted athlete – case study. Kinesiol Slov. 2016;22(1):37–46.

[53] Bussotti M, Magrì D, Previtali E, et al. End-tidal pressure of CO_2_ and exercise performance in healthy subjects. Eur J Appl Physiol. 2008;103(6):727–732. 18521623 10.1007/s00421-008-0773-z

[54] Currell K, Jeukendrup AE. Superior endurance performance with ingestion of multiple transportable carbohydrates. Med Sci Sports Exerc. 2008;40(2):275–281. 18202575 10.1249/mss.0b013e31815adf19

[55] Coggan AR, Swanson SC. Nutritional manipulations before and during endurance exercise: effects on performance. Med Sci Sports Exerc. 1992;24(9 Suppl):S331–S335. 1406206

[56] Hargreaves M, Hawley JA, Jeukendrup A. Pre-exercise carbohydrate and fat ingestion: effects on metabolism and performance. J Sports Sci. 2004;22(1):31–38. 14971431 10.1080/0264041031000140536

[57] Zinn C, Wood M, Williden M, et al. Ketogenic diet benefits body composition and well-being but not performance in a pilot case study of New Zealand endurance athletes. J Int Soc Sports Nutr. 2017;14:22. 28706467 10.1186/s12970-017-0180-0 PMC5506682

[58] McSwiney FT, Wardrop B, Hyde PN, et al. Keto-adaptation enhances exercise performance and body composition responses to training in endurance athletes. Metabolism. 2018;81:25–34. 29108901 10.1016/j.metabol.2017.10.010

[59] Lightowler H, Schweitzer L, Theis S, et al. Changes in weight and substrate oxidation in overweight adults following isomaltulose intake during a 12-week weight loss intervention: a randomized, double-blind, controlled trial. Nutrients. 2019;11(10):2367. 31590285 10.3390/nu11102367 PMC6836138

[60] Klement RJ, Frobel T, Albers T, et al. A pilot case study on the impact of a self-prescribed ketogenic diet on biochemical parameters and running performance in healthy and physically active individuals. Nutr Med. 2013;1(1):10.

[61] Volek JS, Freidenreich DJ, Saenz C, et al. Metabolic characteristics of keto-adapted ultra-endurance runners. Metabolism. 2016;65(3):100–110. 26892521 10.1016/j.metabol.2015.10.028

[62] Buch A, Yeshurun S, Cramer T, et al. The effects of metabolism tracker device (Lumen®) usage on metabolic control in adults with prediabetes: pilot clinical trial. Obes Facts. 2023;16(1):53–61. 10.1159/000527227 PMC9889724 36195053

